# Supplementary material for: DeeDeeExperiment: building an infrastructure for integrating and managing omics data analysis results in R/Bioconductor
Source: Bioinformatics. 2026 Mar 27;42(4):btag157. doi: 10.1093/bioinformatics/btag157 (PMC13253565; doi:10.1093/bioinformatics/btag157)
Supplement: btag157_Supplementary_Data [file btag157_supplementary_data.zip › DeeDeeExperiment - Rev1 - SupplementaryFile.pdf]

Supplementary Information for the `DeeDeeExperiment` package

Bioinformatics Group, IMBEI, University Medical Center Mainz

AUTHORS

Najla Abassi — Institute of Medical Biostatistics, Epidemiology and Informatics (IMBEI), Mainz, Germany

Lea Schwarz — Institute of Medical Biostatistics, Epidemiology and Informatics (IMBEI), Mainz, Germany

Edoardo Filippi — Institute of Medical Biostatistics, Epidemiology and Informatics (IMBEI), Mainz, Germany

Federico Marini — Institute of Medical Biostatistics, Epidemiology and Informatics (IMBEI), Mainz, Germany

PUBLISHED

March 9, 2026

In this document, we demonstrate a concrete example of how *DeeDeeExperiment* improves on common list-based approaches for storing Differential Expression (DEA) and Functional Enrichment (FEA) analyses of omics data by providing a standardized structure to manage these analysis results as well as preserving additional metadata related to the analysis and its context.

In this example we are using a publicly available dataset from Alasoo, et al. "Shared genetic effects on chromatin and gene expression indicate a role for enhancer priming in immune response", published in Nature Genetics, January 2018 (Alasoo et al. 2018) .

The data is made available via the *macrophage* Bioconductor package, which contains the files output from the Salmon quantification (version 0.12.0, with GENCODE v29 reference), as well as the values summarized at the gene level, which we will use to exemplify.

In the `macrophage` experimental setting, the samples are available from 6 different donors, in 4 different conditions (naive, treated with Interferon gamma, with SL1344, or with a combination of Interferon gamma and SL1344).

Let's start by loading all the necessary packages:

```
library("macrophage")
library("DESeq2")
library("mosdef")
library("topGO")
library("org.Hs.eg.db")
```

For demonstration purposes, we will perform DEA using the `DESeq2` framework.

1 Managing bulk RNA-seq analysis results using ad hoc lists

1.1 Differential Expression Analysis (DEA) with *DESeq2*

```
# load data
data(gse, package = "macrophage")
```

```
# set up design
dds_macrophage <- DESeqDataSet(gse, design = ~ line + condition)
rownames(dds_macrophage) <- substr(rownames(dds_macrophage), 1, 15)
keep <- rowSums(counts(dds_macrophage) >= 10) >= 6

dds_macrophage <- dds_macrophage[keep, ]
```

```
dds_macrophage
```

```
#> class: DESeqDataSet
#> dim: 17806 24
#> metadata(7): tximetaInfo quantInfo ... txdbInfo version
#> assays(3): counts abundance avgTxLength
#> rownames(17806): ENSG00000000003 ENSG00000000419 ... ENSG00000285982
#>   ENSG00000285994
#> rowData names(2): gene_id SYMBOL
#> colnames(24): SAMEA103885102 SAMEA103885347 ... SAMEA103885308
#>   SAMEA103884949
#> colData names(15): names sample_id ... condition line
```

```r
# run DESeq
dds_macrophage <- DESeq(dds_macrophage)
# contrasts
resultsNames(dds_macrophage)
```

```
#> [1] "Intercept"                      "line_eiwy_1_vs_diku_1"
#> [3] "line_fikt_3_vs_diku_1"          "line_ieki_2_vs_diku_1"
#> [5] "line_podx_1_vs_diku_1"          "line_qaqx_1_vs_diku_1"
#> [7] "condition_IFNg_vs_naive"        "condition_IFNg_SL1344_vs_naive"
#> [9] "condition_SL1344_vs_naive"
```

```r
# set contrasts of interest
contrast_list <- c("condition_IFNg_vs_naive",
                   "condition_SL1344_vs_naive",
                   "condition_IFNg_SL1344_vs_naive")
```

Now we can extract the DEA results for the contrasts of interest. For this, we create a function that will extract the results and insert them into a list of contrasts. In this example we will call this list `myresuSet`.

```r
# initiate a list to store all the results
myresuSet <- list()
```

We will store DEA and FEA results in `myresuSet` according to the structure shown below:

```
myresuSet
└── id_contrast_1
    ├── res_DESeq     ## The DESeqResults object
    ├── tbl_res_DE    ## A data frame object, with the DE sorted results
    ├── topGO_tbl     ## A data frame object, with the topGO enrichment results in tabular
form
    ├── maplot_res    ## A ggplot object, storing the MA plot for this contrast
└── id_contrast_2
    ├── res_DESeq
    ├── tbl_res_DE
    ├── topGO_tbl
    ├── maplot_res
└── id_contrast_3
    ├── res_DESeq
    ├── tbl_res_DE
    ├── topGO_tbl
    ├── maplot_res
...
```

```r
# a function to generate all results and store it in a list
alltheresults <- function(resuSet, dds_obj, contrast, FDR) {
  id_contrast <- contrast
  resuSet[[id_contrast]] <- list()
  mycoef <- contrast
  message("Extracting results...")
  resuSet[[id_contrast]][["res_DESeq"]] <- results(dds_obj,name = mycoef,
                                                    alpha = FDR)
  message("Performing LFC shrinkage...")
  resuSet[[id_contrast]][["res_DESeq"]] <- lfcShrink(
    dds_obj,coef = mycoef,res = resuSet[[id_contrast]][["res_DESeq"]],
    type = "apeglm"
    )
  resuSet[[id_contrast]][["res_DESeq"]]$SYMBOL <- rowData(dds_obj)$SYMBOL[match(rownames(resuS
  rownames(dds_obj))]
  message("Summary MAplot...")
  summary(resuSet[[id_contrast]][["res_DESeq"]])
  resuSet[[id_contrast]][["maplot_res"]] <-
    ideal::plot_ma(resuSet[[id_contrast]][["res_DESeq"]], ylim = c(-2,2), title = id_contrast,
  message("Extracting DEtables...")
  resuSet[[id_contrast]][["tbl_res_DE"]] <- as.data.frame(
    resuSet[[id_contrast]][["res_DESeq"]]
    )
  resuSet[[id_contrast]][["tbl_res_DE"]] <- cbind(
    rownames(resuSet[[id_contrast]][["tbl_res_DE"]]),
    resuSet[[id_contrast]][["tbl_res_DE"]])
  names(resuSet[[id_contrast]][["tbl_res_DE"]])[1] <- "id"
  resuSet[[id_contrast]][["tbl_res_DE"]]$id <- as.character(
    resuSet[[id_contrast]][["tbl_res_DE"]]$id
    )
  resuSet[[id_contrast]][["tbl_res_DE"]] <- resuSet[[id_contrast]][["tbl_res_DE"]][order(
    resuSet[[id_contrast]][["tbl_res_DE"]]$padj
    ), ]
  if (!is.null(FDR))
    resuSet[[id_contrast]][["tbl_res_DE"]] <- resuSet[[id_contrast]][["tbl_res_DE"]][!(is.na(r
  return(resuSet)
}
```

```
# set FDR
FDR = 0.05
```

```
# extract results and save them into a list

for (contrast_id in contrast_list) {
  myresuSet <- alltheresults(myresuSet, dds_macrophage,
                             contrast = contrast_id,
                             FDR = FDR)
}
```

```
#> 
#> out of 17806 with nonzero total read count
#> adjusted p-value < 0.05
#> LFC > 0 (up)       : 3192, 18%
#> LFC < 0 (down)     : 2887, 16%
#> outliers [1]       : 0, 0%
#> low counts [2]     : 0, 0%
#> (mean count < 3)
#> [1] see 'cooksCutoff' argument of ?results
#> [2] see 'independentFiltering' argument of ?results

#> 
#> out of 17806 with nonzero total read count
#> adjusted p-value < 0.05
#> LFC > 0 (up)       : 5282, 30%
#> LFC < 0 (down)     : 4785, 27%
#> outliers [1]       : 0, 0%
#> low counts [2]     : 0, 0%
#> (mean count < 3)
#> [1] see 'cooksCutoff' argument of ?results
#> [2] see 'independentFiltering' argument of ?results

#> 
#> out of 17806 with nonzero total read count
#> adjusted p-value < 0.05
#> LFC > 0 (up)       : 6526, 37%
#> LFC < 0 (down)     : 5956, 33%
#> outliers [1]       : 0, 0%
#> low counts [2]     : 0, 0%
#> (mean count < 3)
#> [1] see 'cooksCutoff' argument of ?results
#> [2] see 'independentFiltering' argument of ?results
```

Now we have `myresuSet`, a list with DE results per contrast.

As it is illustrated here, this approach gives the complete freedom to add any object from the environment as list elements, but completely lacks a set of validation methods to consolidate the final object.

Now let's add the FEA results.

1.2 Functional Enrichment Analysis (FEA) with *topGO*

```
for (contrast_id in contrast_list) {
  if (nrow(myresuSet[[contrast_id]][["res_DESeq"]]) > 0) {
  myresuSet[[contrast_id]][["topGO_tbl"]] <-
    run_topGO(
              de_genes = myresuSet[[contrast_id]][["tbl_res_DE"]]$SYMBOL,
              bg_genes = rowData(dds_macrophage)$SYMBOL,
              ontology = "BP",
              mapping = "org.Hs.eg.db",
              add_gene_to_terms = TRUE,
              gene_id = "symbol")
  }
}
```

Now we have our results ready to explore.

```
str(myresuSet)
```

```
#>         optional_aes:
#>         parameters: function
#>         required_aes:
#>         retransform: TRUE
#>         setup_data: function
#>         setup_params: function
#>         super:  <ggproto object: Class Stat, gg>
#>     stat_params: list
#>     super:  <ggproto object: Class Layer, gg>
#>  .. .. .. $ geom_rug  :Classes 'LayerInstance', 'Layer', 'ggproto', 'gg' <ggproto object:
Class LayerInstance, Layer, gg>
#>     aes_params: list
#>     compute_aesthetics: function
#>     compute_geom_1: function
#>     compute_geom_2: function
```

As you can see, all results are stored, but navigating this structure can be difficult, and displaying them in a readable way can be cumbersome.

In practice, this often leads users to extract and store result tables separately, e.g. in Excel sheets. Unfortunately, with this approach results are more prone to being accidentally deleted, overwritten, or stripped of their original context …

```
# oops 1, deleting results
myresuSet$condition_IFNg_vs_naive$res_DESeq <- NULL
```

```
# oops 2, overwriting results
myresuSet$condition_IFNg_vs_naive$res_DESeq <- dds_macrophage
```

In addition, the original count matrix and its metadata cannot be accessed if we are only saving the results. So the context is lost and these results cannot be reproduced unless one gets the counts.

2 Managing bulk RNA-seq analysis results using `DeeDeeExperiment`

With *DeeDeeExperiment* , DEA and FEA results are stored together with the original object and relevant analysis metadata within a single standardized container that can be readily shared and used within the Bioconductor ecosystem. By extending the *SingleCellExperiment* class, `DeeDeeExperiment` enforces a consistent internal structure in which results are explicitly associated with named contrasts, analysis metadata, software versions, and contextual information. These guarantees reduce the risk of silent errors, loss of provenance, and ambiguity when sharing or revisiting analyses.

```
library("DeeDeeExperiment")
```

Let's create a list of DEA and FEA results per contrast and give each contrast a meaningful name.

```
# dea
de_list <- list(
  IFNg_vs_naive = myresuSet$condition_IFNg_vs_naive$res_DESeq,
  SL1344_vs_naive = myresuSet$condition_SL1344_vs_naive$res_DESeq
  )
# fea
fe_list <- list(
  IFNg_vs_naive = myresuSet$condition_IFNg_vs_naive$topGO_tbl,
  SL1344_vs_naive = myresuSet$condition_SL1344_vs_naive$topGO_tbl
  )
```

Now let's assemble our `dde` object and display it

```
dde <- DeeDeeExperiment(sce = dds_macrophage, # original dds object used to
                        #perform the analysis
                        de_results = de_list, # dea list
                        enrich_results = fe_list) # fea list

dde
```

```
#> class: DeeDeeExperiment
#> dim: 17806 24
#> metadata(8): tximetaInfo quantInfo ... version singlecontrast
#> assays(7): counts abundance ... H cooks
#> rownames(17806): ENSG00000000003 ENSG00000000419 ... ENSG00000285982
#>   ENSG00000285994
#> rowData names(58): gene_id SYMBOL ... SL1344_vs_naive_pvalue
#>   SL1344_vs_naive_padj
#> colnames(24): SAMEA103885102 SAMEA103885347 ... SAMEA103885308
#>   SAMEA103884949
#> colData names(15): names sample_id ... condition line
#> reducedDimNames(0):
```

```
#> mainExpName: NULL
#> altExpNames(0):
#> dea(2): IFNg_vs_naive, SL1344_vs_naive
#> fea(2): IFNg_vs_naive, SL1344_vs_naive
```

Alternatively, the `dde` object can be initialized directly from the `dds` object and populated later

```
dde_built_step_by_step <- DeeDeeExperiment(sce = dds_macrophage)
dde_built_step_by_step
```

```
#> class: DeeDeeExperiment
#> dim: 17806 24
#> metadata(7): tximetaInfo quantInfo ... txdbInfo version
#> assays(7): counts abundance ... H cooks
#> rownames(17806): ENSG00000000003 ENSG00000000419 ... ENSG00000285982
#>   ENSG00000285994
#> rowData names(52): gene_id SYMBOL ... deviance maxCooks
#> colnames(24): SAMEA103885102 SAMEA103885347 ... SAMEA103885308
#>   SAMEA103884949
#> colData names(15): names sample_id ... condition line
#> reducedDimNames(0):
#> mainExpName: NULL
#> altExpNames(0):
#> dea(0):
#> fea(0):
```

```
# add DEA results
# N.B: results can also be passed as a named list
dde_built_step_by_step <- addDEA(dde_built_step_by_step,
                                 dea = myresuSet$condition_IFNg_vs_naive$res_DESeq)

dde_built_step_by_step <- addDEA(dde_built_step_by_step,
                                 dea = myresuSet$condition_SL1344_vs_naive$res_DESeq)

dde_built_step_by_step
```

```
#> class: DeeDeeExperiment
#> dim: 17806 24
#> metadata(8): tximetaInfo quantInfo ... version singlecontrast
#> assays(7): counts abundance ... H cooks
#> rownames(17806): ENSG00000000003 ENSG00000000419 ... ENSG00000285982
#>   ENSG00000285994
#> rowData names(58): gene_id SYMBOL ...
#>   myresuSet$condition_SL1344_vs_naive$res_DESeq_pvalue
#>   myresuSet$condition_SL1344_vs_naive$res_DESeq_padj
#> colnames(24): SAMEA103885102 SAMEA103885347 ... SAMEA103885308
#>   SAMEA103884949
#> colData names(15): names sample_id ... condition line
#> reducedDimNames(0):
#> mainExpName: NULL
#> altExpNames(0):
#> dea(2): myresuSet$condition_IFNg_vs_naive$res_DESeq,
myresuSet$condition_SL1344_vs_naive$res_DESeq
#> fea(0):
```

```
# rename contrast to keep context
dde_built_step_by_step <- renameDEA(dde_built_step_by_step,
                                    old = c("myresuSet$condition_IFNg_vs_naive$res_DESeq",
                                            "myresuSet$condition_SL1344_vs_naive$res_DESeq"),
                                    new = c("condition_IFNg_vs_naive",
                                            "condition_SL1344_vs_naive"))
```

```
# add FEA results
dde_built_step_by_step <- addFEA(dde_built_step_by_step,
                                 fea = list(
  condition_IFNg_vs_naive = myresuSet$condition_IFNg_vs_naive$topGO_tbl,
  condition_SL1344_vs_naive = myresuSet$condition_SL1344_vs_naive$topGO_tbl
  ))
dde_built_step_by_step
```

```
#> class: DeeDeeExperiment
#> dim: 17806 24
#> metadata(8): tximetaInfo quantInfo ... version singlecontrast
#> assays(7): counts abundance ... H cooks
#> rownames(17806): ENSG00000000003 ENSG00000000419 ... ENSG00000285982
#>   ENSG00000285994
#> rowData names(58): gene_id SYMBOL ... condition_SL1344_vs_naive_pvalue
#>   condition_SL1344_vs_naive_padj
#> colnames(24): SAMEA103885102 SAMEA103885347 ... SAMEA103885308
#>   SAMEA103884949
#> colData names(15): names sample_id ... condition line
#> reducedDimNames(0):
```

```
#> mainExpName: NULL
#> altExpNames(0):
#> dea(2): condition_IFNg_vs_naive, condition_SL1344_vs_naive
#> fea(2): condition_IFNg_vs_naive, condition_SL1344_vs_naive
```

```
# get a quick summary on the analysis
summary(dde_built_step_by_step)
```

```
#> DE Results Summary:
#>                    DEA_name   Up Down  FDR
#>    condition_IFNg_vs_naive 3192 2887 0.05
#>  condition_SL1344_vs_naive 5282 4785 0.05
#>
#> FE Results Summary:
#>                    FEA_Name                 Linked_DE FE_Type Term_Number
#>    condition_IFNg_vs_naive   condition_IFNg_vs_naive   topGO        6091
#>  condition_SL1344_vs_naive condition_SL1344_vs_naive   topGO        6091
```

Now we can save this object that has recorded everything for us, and can be shared without losing information.

```
saveRDS(dde, "dde_macrophage.RDS")
```

At a later time, we can load the object and still access everything related to the analysis

```
# get available contrasts
getDEANames(dde)
```

```
#> [1] "IFNg_vs_naive"   "SL1344_vs_naive"
```

```
# access dea and fea results
de_IFNg_vs_naive <- getDEA(dde,"IFNg_vs_naive", format = "minimal")
fe_IFNg_vs_naive <- getFEA(dde, "IFNg_vs_naive", format = "minimal")

# get a quick summary on the analysis
summary(dde)
```

```
#> DE Results Summary:
#>          DEA_name   Up Down  FDR
#>    IFNg_vs_naive 3192 2887 0.05
#>  SL1344_vs_naive 5282 4785 0.05
#>
#> FE Results Summary:
#>          FEA_Name       Linked_DE FE_Type Term_Number
#>    IFNg_vs_naive   IFNg_vs_naive   topGO        6091
#>  SL1344_vs_naive SL1344_vs_naive   topGO        6091
```

```
# we still have the original object and metadata about the analysis, in case you
# come back 1 month later and forgot what you did
original_de_IFNg_vs_naive <- getDEA(dde,"IFNg_vs_naive", format = "original")

# and of course we store for each contrast, metadata such as the packages and
# versions used in this analysis, lfc threshold ...
getDEAInfo(dde)[["IFNg_vs_naive"]]
```

```
#> $alpha
#> [1] 0.05
#>
#> $lfcThreshold
#> [1] 0
#>
#> $metainfo_logFC
#> [1] "log2 fold change (MAP): condition IFNg vs naive"
#>
#> $metainfo_pvalue
#> [1] "Wald test p-value: condition IFNg vs naive"
#>
#> $original_object
#> $original_object$metadata_storage
#> [1] "singlecontrast"
#>
#> $original_object$key
#> [1] "IFNg_vs_naive"
#>
#> $original_object$coef
#> NULL
#>
#>
#> $package
#> [1] "DESeq2"
#>
```

```
#> $package_version
#> [1] '1.51.6'
```

These information are stored within the object and preserved when saving and reloading the `dde` object, supporting reproducibility across time and collaborators.

By extending `SingleCellExperiment`, `DeeDeeExperiment` adheres to Bioconductor's established standards for interoperability, rigorous testing, and documentation, enabling seamless integration with existing downstream tools and workflows for analysis, interpretation, and interactive exploration.

```
# for e.g. we can seamlessly insert a dde in iSEE and start the interactive
# visualization of the object
library("iSEE")
iSEE::iSEE(dde)
```

For more details and methods and other use cases of `DeeDeeExperiment`, please check the package's original vignette and the preprint

Session info

```
sessionInfo()
```

```
#> R Under development (unstable) (2026-02-10 r89394)
#> Platform: x86_64-apple-darwin20
#> Running under: macOS Sequoia 15.7.3
#> 
#> Matrix products: default
#> BLAS:   /Library/Frameworks/R.framework/Versions/4.6-x86_64/Resources/lib/libRblas.0.dylib
#> LAPACK: /Library/Frameworks/R.framework/Versions/4.6-
x86_64/Resources/lib/libRlapack.dylib;  LAPACK version 3.12.1
#> 
#> locale:
#> [1] en_US.UTF-8/en_US.UTF-8/en_US.UTF-8/C/en_US.UTF-8/en_US.UTF-8
#> 
#> time zone: Europe/Berlin
#> tzcode source: internal
#> 
#> attached base packages:
#> [1] stats4    stats     graphics  grDevices utils     datasets  methods
#> [8] base
#> 
#> other attached packages:
#>  [1] DeeDeeExperiment_1.1.4      SingleCellExperiment_1.33.0
#>  [3] org.Hs.eg.db_3.22.0         topGO_2.63.0
#>  [5] SparseM_1.84-2              GO.db_3.22.0
#>  [7] AnnotationDbi_1.73.0        graph_1.89.1
#>  [9] mosdef_1.7.0                DESeq2_1.51.6
#> [11] SummarizedExperiment_1.41.1 Biobase_2.71.0
#> [13] MatrixGenerics_1.23.0       matrixStats_1.5.0
#> [15] GenomicRanges_1.63.1        Seqinfo_1.1.0
#> [17] IRanges_2.45.0              S4Vectors_0.49.0
#> [19] BiocGenerics_0.57.0         generics_0.1.4
#> [21] macrophage_1.27.0
#> 
#> loaded via a namespace (and not attached):
#>   [1] fs_1.6.6                 bitops_1.0-9             enrichplot_1.31.0
#>   [4] httr_1.4.7               webshot_0.5.5            RColorBrewer_1.1-3
#>   [7] Rgraphviz_2.55.0         numDeriv_2016.8-1.1      tools_4.6.0
#>  [10] R6_2.6.1                 DT_0.34.0                lazyeval_0.2.2
#>  [13] mgcv_1.9-4               apeglm_1.33.0            withr_3.0.2
#>  [16] prettyunits_1.2.0        gridExtra_2.3            fdrtool_1.2.18
#>  [19] cli_3.6.5                TSP_1.2.6                slam_0.1-55
#>  [22] mvtnorm_1.3-3            S7_0.2.1                 genefilter_1.93.0
#>  [25] goseq_1.63.0             Rsamtools_2.27.0         systemfonts_1.3.1
#>  [28] yulab.utils_0.2.4        gson_0.1.0               txdbmaker_1.7.3
#>  [31] DOSE_4.5.0               rentrez_1.2.4            AnnotationForge_1.53.0
#>  [34] dichromat_2.0-0.1        bbmle_1.0.25.1           limma_3.67.0
#>  [37] rstudioapi_0.18.0        RSQLite_2.4.6            GOstats_2.77.0
#>  [40] gridGraphics_0.5-1       BiocIO_1.21.0            gtools_3.9.5
#>  [43] dplyr_1.2.0              dendextend_1.19.1        Matrix_1.7-4
#>  [46] abind_1.4-8              lifecycle_1.0.5          edgeR_4.9.2
#>  [49] yaml_2.3.12              gplots_3.3.0             qvalue_2.43.0
#>  [52] SparseArray_1.11.10      BiocFileCache_3.1.0      grid_4.6.0
#>  [55] blob_1.3.0               promises_1.5.0           crayon_1.5.3
#>  [58] bdsmatrix_1.3-7          shinydashboard_0.7.3     ggtangle_0.1.1
#>  [61] lattice_0.22-9           cowplot_1.2.0            annotate_1.89.0
#>  [64] GenomicFeatures_1.63.1   cigarillo_1.1.0          KEGGREST_1.51.1
#>  [67] pillar_1.11.1            knitr_1.51               fgsea_1.37.4
#>  [70] rjson_0.2.23             codetools_0.2-20         fastmatch_1.1-8
#>  [73] glue_1.8.0               ggiraph_0.9.4            ggfun_0.2.0
#>  [76] fontLiberation_0.1.0     data.table_1.18.2.1      vctrs_0.7.1
```

```
#>  [79] png_0.1-8               treeio_1.35.0          gtable_0.3.6
#>  [82] assertthat_0.2.1        emdbook_1.3.14         cachem_1.1.0
#>  [85] xfun_0.56               S4Arrays_1.11.1        mime_0.13
#>  [88] coda_0.19-4.1           survival_3.8-6         pheatmap_1.0.13
#>  [91] seriation_1.5.8         iterators_1.0.14       statmod_1.5.1
#>  [94] Category_2.77.0         nlme_3.1-168           ggtree_4.1.1
#>  [97] bit64_4.6.0-1           fontquiver_0.2.1       progress_1.2.3
#> [100] filelock_1.0.3          UpSetR_1.4.0           GenomeInfoDb_1.47.2
#> [103] KernSmooth_2.23-26      otel_0.2.0             DBI_1.2.3
#> [106] tidyselect_1.2.1        bit_4.6.0              compiler_4.6.0
#> [109] curl_7.0.0              httr2_1.2.2            BiasedUrn_2.0.12
#> [112] fontBitstreamVera_0.1.1 DelayedArray_0.37.0    plotly_4.12.0
#> [115] rtracklayer_1.71.3      scales_1.4.0           caTools_1.18.3
#> [118] RBGL_1.87.0             rappdirs_0.3.4         stringr_1.6.0
#> [121] digest_0.6.39           shinyBS_0.63.0         rmarkdown_2.30
#> [124] ca_0.71.1               XVector_0.51.0         htmltools_0.5.9
#> [127] pkgconfig_2.0.3         base64enc_0.1-6        lpsymphony_1.39.0
#> [130] ideal_2.5.0             dbplyr_2.5.1           fastmap_1.2.0
#> [133] rlang_1.1.7             htmlwidgets_1.6.4      UCSC.utils_1.7.1
#> [136] shiny_1.12.1            farver_2.1.2           IHW_1.39.0
#> [139] jsonlite_2.0.0          BiocParallel_1.45.0    GOSemSim_2.37.2
#> [142] RCurl_1.98-1.17         magrittr_2.0.4         ggplotify_0.1.3
#> [145] patchwork_1.3.2         Rcpp_1.1.1             ape_5.8-1
#> [148] viridis_0.6.5           gdtools_0.5.0          rintrojs_0.3.4
#> [151] stringi_1.8.7           MASS_7.3-65            plyr_1.8.9
#> [154] parallel_4.6.0          ggrepel_0.9.6          Biostrings_2.79.4
#> [157] splines_4.6.0           hms_1.1.4              geneLenDataBase_1.47.0
#> [160] locfit_1.5-9.12         igraph_2.2.1           reshape2_1.4.5
#> [163] biomaRt_2.67.1          XML_3.99-0.22          evaluate_1.0.5
#> [166] BiocManager_1.30.27     foreach_1.5.2          tweenr_2.0.3
#> [169] httpuv_1.6.16           tidyr_1.3.2            purrr_1.2.1
#> [172] polyclip_1.10-7         heatmaply_1.6.0        ggplot2_4.0.2
#> [175] ggforce_0.5.0           xtable_1.8-4           restfulr_0.0.16
#> [178] tidytree_0.4.7          later_1.4.5            viridisLite_0.4.3
#> [181] tibble_3.3.1            clusterProfiler_4.19.1 aplot_0.2.9
#> [184] memoise_2.0.1           registry_0.5-1         GenomicAlignments_1.47.0
#> [187] writexl_1.5.4           GSEABase_1.73.0        shinyAce_0.4.4
#> [190] BiocStyle_2.39.0
```

References

Alasoo, Kaur, Julia Rodrigues, Subhankar Mukhopadhyay, Andrew J. Knights, Alice L. Mann, Kousik Kundu, Christine Hale, Gordon Dougan, and Daniel J. Gaffney. 2018. "Shared genetic effects on chromatin and gene expression indicate a role for enhancer priming in immune response." *Nature Genetics* 50 (3): 424–31. https://doi.org/10.1038/s41588-018-0046-7.
